# Factors affecting the prolongation of mechanical ventilation in patients after cardiac surgery

**DOI:** 10.1186/s13019-024-03247-z

**Published:** 2025-01-29

**Authors:** Mahdieh Sharifzadeh Kermani, Tania Dehesh, Shiva Pouradeli, Bahareh Soltani Esmaili

**Affiliations:** 1 Clinical Research Development Unit, Shafa Hospital, Kerman University of Medical Sciences, Kerman, Iran; 2 Modeling in Health Research Center, Institue for Futures Studies in Health, Kerman University of Medical Sciences, Kerman, Iran

**Keywords:** Mechanical ventilation, Cardiac surgical procedures, Respiratory failure, Critical care, Postoperative complication

## Abstract

**Background:**

This study aimed to investigate the major predictive factors associated with prolonged mechanical ventilation(PMV) following cardiac surgery.

**Methods:**

This retrospective, cross-sectional, descriptive-analytical study was conducted from September 2021 to March 2022, involving 244 patients who underwent cardiac surgery. PMV was defined as mechanical ventilation for more than 24 h. Potential risk factors before, during, and after surgery were examined and recorded. Logistic regression analysis was performed to assess the relationship between demographic, clinical variables, and prolonged mechanical ventilation. A significance level of 0.05 was used for data analysis.

**Results:**

The study population consisted of 68.4% male and 31.6% female patients, with 86.9% undergoing CABG surgery. PMV was observed in 13.1% of the patients. The findings revealed that the incidence of postoperative pneumonia increased the likelihood of PMV by more than 7 times [OR = 7.24, 95% CI=(5.12,8.14), P-value = 0.001]. Similarly, respiratory failure was associated with a 7.5-fold increase in the odds of PMV [OR = 7.56, 95% CI=(4.48,8.77), P-value = 0.042]. Drainage of one liter of blood on the first postoperative day increased the risk of PMV by 2.2 times [OR = 2.21, 95% CI=(1.98,2.46), P-value = 0.032], and the use of epinephrine was associated with a 2.73-fold increase in the odds [OR = 2.73, 95% CI=(2.24,3.11), P-value = 0.022]. Risk of PMV in the patients who had cardiac dysfunctin increased by more than 2 times.[OR = 2.58, 95%; CI = (1.33.2.87); P-value = 0.042]. In the patients need an Intra Aortic Balloon Pump(IABP) risk of PMV increased by more than 2 times. (OR = 2.74,95%,CI = 1.36,5.47: Pvalue = 0.03). The risk of PMV in the patients who had cerebrovascular accident(CVA) increased by more than three times [OR = 3.75, 95% CI = 1.26,4.84; P-value = 0.044]. For each unit increase in Euro Score 2 the chance of PMV increased by 1.38 TIMES. Furthermore ICU Mortality had a significant relationship with PMV.(Pvalue < 0.001).

**Conclusion:**

The study identified postoperative complications, such as pneumonia, respiratory failure, high drainage, need to an IABP, higher EURO Score 2, Cardiac dysfunction, CVA and the use of epinephrine, as independent risk factors for PMV following cardiac surgery.

## Background

Patients who undergo cardiac surgery are at risk of postoperative complications. Most of these patients have advanced heart disease along with some co-morbidities that can be problematic. Prolonged mechanical ventilation (PMV) is a common complication after cardiac surgery. Although the exact definition of prolonged mechanical ventilation is debated, according to STS, a period more than 24 h after surgery is considered prolonged [1, 2]. According to previous studies, the incidence of prolonged mechanical ventilation after surgery was 6.2–15% [2–5].

Prolonged mechanical ventilation is a multifactorial phenomenon. Patient characteristics and underlying diseases, the type and severity of the surgery, and some demographic and medical variables before the operation are effective at the time of tracheal tube removal, including age, chronic renal failure, chronic obstructive pulmonary disease (COPD), obesity, congestive heart failure before surgery, relative oxygen pressure before surgery, decrease in forced expiratory volume in 1 s, history of transient ischemic attack or brain stroke before surgery, left ventricular ejection fraction less than 30%, acute respiratory distress syndrome, and neurological disorders [3, 5]. Furthermore, patients who needed long-term use of mechanical ventilation experienced various complications such as intrapulmonary shunt, pneumonia, atelectasis, sepsis, endocarditis, gastrointestinal bleeding, stroke, renal failure, or deep wound infection in the chest area, and a higher mortality rate has been reported in these patients [4, 6].

Attempts to reduce postoperative ventilation time may have medical and financial benefits. However, early extubation in in a situation where the patient is still at risk of hypothermia, hemorrhage, and cardiorespiratory instability may sometimes outweigh the potential benefits and increase the likelihood of ICU readmission. Evidence has shown that re-intubation compared to the first time, increases the risk of hospital-acquired pneumonia by 8 times and mortality by 6–12 times, and thus the interruption of mechanical ventilation should be planned [7, 8].

Although some parameters can predict PMV, they have not yet been confirmed [4] Given the various parameters identified as risk factors in different studies, we opted to conduct a comprehensive review of potential risk factors for long-term ventilation at our center. This review aims to identify these factors early to facilitate timely prevention and treatment. The findings from this study can help in modifying and addressing these potential risk factors effectively.

## Methods

### Study design and setting

This was a retrospective, cross-sectional, descriptive-analytical study conducted at Shafa Hospital in Kerman, southern Iran, from September 2021 to March 2022. The study population included all 244 patients who underwent cardiac surgery during the study period.

## Data collection

Patient data were collected by reviewing their medical records and the hospital’s filing system using a standardized data collection form. The following information was gathered:

**Demographic characteristics**: age, gender, smoking status, opioid use, body mass index.

**Clinical characteristics**: ejection fraction, comorbidities, type of cardiac surgery, duration of surgery, use and duration of cardiopulmonary bypass, NYHA Score, European System for Cardiac Operative Risk Evaluation Score(Euro Score 2).

**Postoperative outcomes**: duration of mechanical ventilation, presence of prolonged mechanical ventilation, length of stay in the intensive care unit, use and duration of inotropic support, drainage volume, occurrence of stroke, emergency/elective surgery, use of the IABP, cardiac dysfunction(myocardial ischemia, arytmia, pulmonary edema, decreased EF), pulmonary complications, cerebrovascularaccident, (CVA) and icu mortality.

## Definition of prolonged mechanical ventilation

PMV was defined as the need for mechanical ventilation for more than 24 h after cardiac surgery.

## Inclusion criteria

Any patient who undervent open cardiac surgery including coronary artery.

bypass graft surgery, valve repair or replacement, aortic aneurysm or.

dissection repair, atrial septal defect(ASD) or a combination of.

these, and surgery was performed in an elective, or emergency.

setting.

## Exclusin criteria

Patients wewe excluded from the study if they were pericardiectomy or age < 18 y or if the patients file information was missing a lot.

**Sterategy of mechanical ventilation during and after surgery**:

Mode SIMV/Assist controlled ventilatin.

TV 8 ml/kg of IBW (Ideal body weight).

PEEP 5 (consider 7–8 in obese patients and in icu according to patient to set ParteriaO2 > 60 mmhg).

RR 12–20 (titrate for EtCO2 30–40).

FiO2 to keep oxygen saturation > 92% (90% for severe.

COPD patients)

Set tv to keep pressure of peek < 40 Cm H2o and Platu pressure < 30 Cm H2o.

Send ABG (point of care).

**Flowchart. 1:**
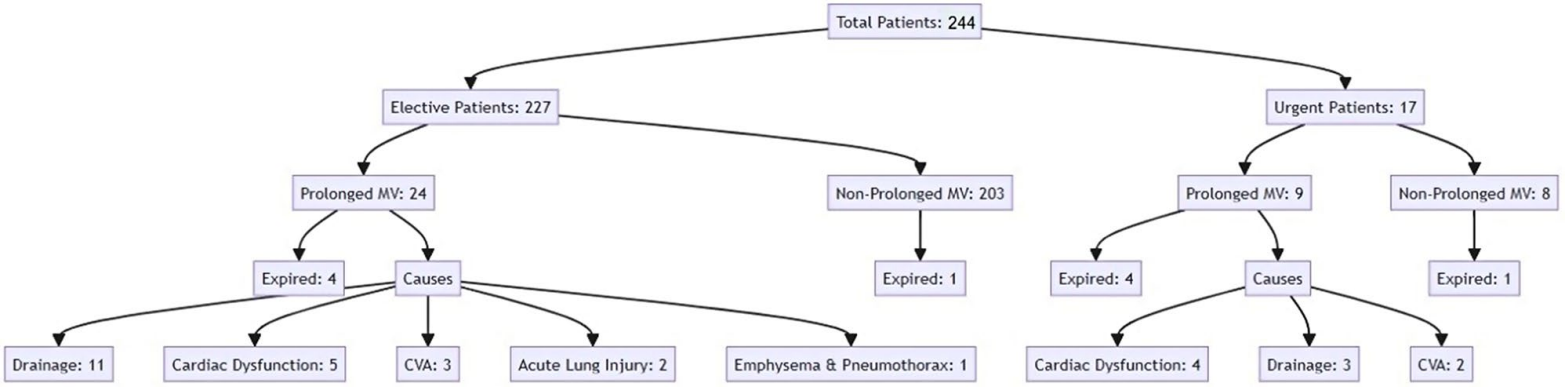
Diagram of patient groups

### Data analysis

Descriptive statistics were used to summarize the data, with qualitative variables presented as frequencies and percentages, and quantitative variables as means and standard deviations. To compare the demographic variables between the two groups (Table 1), we first assessed the normality of the data distribution. In cases where normality was not achieved, we utilized nonparametric tests instead of parametric tests.

Univariate logistic regression analysis was performed to examine the relationship between demographic and clinical variables with PMV. Variables with a significance level of ≤ 0.25 in the univariate analysis were then included in a multiple logistic regression model.

Multicollinearity between variables was assessed using the variance inflation factor, and no significant multicollinearity was detected. The final model was selected using a backward selection approach based on the likelihood ratio test and Akaike’s information criteria. Potential interaction effects were checked at a significance level of 0.1, and none were found to be significant. Crude and adjusted odds ratios (ORs) with 95% confidence intervals were reported. The Hosmer-Lemeshow goodness-of-fit test was used to assess the adequacy of the final logistic regression model.Due to the small number of patients with prolonged mechanical ventilation (*n* = 32) compared to those without (*n* = 212), a Firth penalized logistic regression analysis was performed using R software version 4.3.1 with the logistf package. A significance level of 0.05 was considered for the data analysis.

### Consent to participate declaration

This study is a retrospective analysis of existing data. As such, informed consent was not obtained from participants, as the data was collected prior to the initiation of this study and was anonymized. The study was conducted in accordance with ethical standards, and the confidentiality of participant information was strictly maintained throughout the research process.

This retrospective study has received ethical approval under the code IR.KMU.AH.REC.1401.62, ensuring compliance with established ethical standards in research.

## Results

The participants in this study were 244 patients. The data showed that 68.4% of patients were male and 31.6% were female with an average age of 58.75 ± 12.01 (age range of 15–82 years). The mean BMI of the patients was 24.11 ± 0.99. Moreover, 49.6% of the patients had drug addiction. An analysis of the frequency of underlying diseases indicated that 35.7% of the patients had diabetes, 67.6% had hypertension, 7.4% had copd and 4.9% had chronic renal failure. In addition,90.1% (202) of patients had CABG surgery and3.68% (9 patients) had CABG and valve surgery, 9.42%(23 patients) had valve surgery(AVR, MVR, TVR),3.27%(8 pateints) had ASD and 0.40%(1 pateint) aortic dissection and0.40% (1patient) atrial myxoma. Furthermore, 93% of surgeries were elective and 7% were emergency. In emergency surgery 23.5%(4 patients) had cardiac arrest upon arrival.

48.8% of the patients were operated on-pump and 51.2% were off-pump. On the first day after surgery, 84.4% of the patients had less than one liter of drainage and 15.2% had more than one liter of drainage.9.8% of the patients needed a balloon pump. Concerning other risk factors examined, 59% of the patients used blood products. The postoperative complications included pneumonia (11.5%), respiratory failure (4.1%), pleural effusion (11.1%), atlectasis (6.1%),cardiac dysfunction(7%),arytmia (9.5%), stroke (2.5%), acute renal failure (17.6%) and(1.2%) 3 patients underwent dialysis.2.7% of the patients had revision. Overall, 13.1% of the patients had prolonged mechanical ventilation. Complications with less incidence included (1.2%)3cases of sepsis, (0.81%)2 cases of DKA, (0.81%)2 cases of gastrointestinal bleeding.

As can be seen in Table 1, the preoperative variables had no significant relationship with the use of prolonged mechanical ventilation, except for patients undergoing emergency surgeries as these patients used significantly more prolonged mechanical ventilation (P-value < 0.001). **Euro Score 2 had also significant relationship with PMV.(P-value = 0.02)** Moreover, the patients who had a longer blood pump time during surgery used PMV more frequently in the postoperative phase (P-value = 0.036).The patient who needed balloon pump had significant relationship with PMV.(P-VALUE = 0.018) also pneumonia increased prolonged ventilation more than 6 times(P value < 0.001). All postoperative variables, except for receiving packed cells, showed a significant relationship with PMV.

ICU Mortality had a significant relationship with prolonged ventilation.(Pvalue < 0.001). It was 22% in prolonged ventilation group.

The findings in Table 2 indicate that the occurrence of pneumonia, respiratory failure, high drainage, need to balloon pump, higher EURO Score 2,Cardiac dysfunction, the number of days of epinephrine use, and the length of stay in the ICU significantly increased the odds of PMV. For each day of stay in the ICU, the chance of PMV increased by 1.5 times [OR = 1.53, 95%; CI = 1.33,1.75; P-value = 0.003].For each unit increase in Euro Score 2 the chance of PMV increased by 1.38 TIMES. (OR = 1.38,95%;CI = 1.12,1.65).

The occurrence of pneumonia increased the chance of using PMV more than 7 times [OR = 7.24, 95%; CI = 5.12, 8.14; P-value = 0.001]. Respiratory failure increased the chance of PMV by 7.5 times [OR = 7.56, 95% CI = 4.48, 8.77; P-value = 0.042] and drainage of more than one liter of blood on the first day after surgery increased the chance of PMV by 2.2 times [OR = 2.21, 95%; CI = 1.98,2.45; P-value = 0.031]. The risk of PMV in the patients who received vasopressor epinephrine increased by 2.73 times [OR = 2.73, 95% CI = 2.24,3.11; P-value = 0.022] compared to the patients who did not receive this vasopressor. In addition, the risk of PMV in the patients who had cerebrovascular accident increased by more than three times [OR = 3.75, 95% CI = 1.26,4.84; P-value = 0.044]. The findings also revealed that risk of PMV in the patients who had cardiac dysfunctin increased by more than 2 times.[OR = 2.58, 95%; CI = (1.33,2.87); P-value = 0.042]. In the patients need an IABP risk of PMV increased by more than 2 times.(OR = 2.74,95%,CI = 1.36,5.47: Pvalue = 0.03).

**Table 1:** Relationship of demographic and clinical variables (before, during, and after surgery) with prolonged mechanical ventilation after cardiac surgery

Before surgery	Levels	Ventilation more than 24 h	Ventilation less than 24 h	*P*-value
Gender	Male	24(75%)	143(67.5%)	0.392
	Female	8(25%)	69(32.5%)	
BMI (Mean$\:\pm\:$ SD)		24.51 ± 3.64	24.05 ± 4.05	0.538
Age (Mean$\:\pm\:$ SD)		58.22 ± 11.86	60.88 ± 12.88	0.245
EF (Mean ± SD)		44.06 ± 8.93	44.01 ± 10.39	0.976
EURO Score 2		2.09 ± 1.89	1.25 ± 1.03	0.020
Opioid addiction	Yes	20(62.5%)	101(47.6%)	0.117
	No	12(37.5%)	111(52.4%)	
Cigarette smoking	Yes	2(6.3%)	22(10.4%)	0.75
	No	30(93.8%)	190(89.6%)	
**Underlying diseases**				
Diabetes mellitus	Yes	13(40.6%)	74(34.9%)	0.529
	No	19(59.4%)	138(65.1%)	
Hypertension	Yes	23(71.9%)	142(67%)	0.581
	No	9(28.1%)	70(33%)	
CKD	Yes	4(12.5%)	204(95.8%)	0.061
	No	28(87.5%)	8(3.8%)	
Urgency	Yes	9(28.1%)	8(3.8%)	**< 0.001**
	No	23(71.9%)	204(96.2%)	
CABG	Yes	29(90.6%)	183(86.3%)	0.778
	No	3(9.4%)	29(13.7%)	
Copd	Yes	4(12.5%)	14(6.6%)	0.234
	No	28(87.5%)	198(93.4%)	
**During surgery**				
Duration of surgery (Mean ± SD)		260.16 ± 57.96	250.33 ± 64.14	0.281
Using pump	Off	12(37.5%)	113(53.3%)	0.096
	ON	20(62.5%)	99(46.7%)	
Pump-time		46.06 ± 48.73	63.34 ± 17.36	**0.036**
Balloon pump	Yes	8(25%)	17(8%)	**0.018**
	No	24(75%)	195(92%)	
**After surgery**				
ICU admission (day) (Mean$\:\pm\:$ SD)		4.49 ± 2.32	7.69 ± 5.85	**0.002**
ICU Mortality	Yes	7(21.9%)	3(1.4%)	**<** 0.001
	No	25(78.1%)	209(98.6%)	
Pneumonia	Yes	14(43.8%)	14(6.6%)	**< 0.001**
	No	18(56.3%)	198(93.4%)	
Respiratory Failure	Yes	7(21.9%)	3(1.4%)	**< 0.001**
	No	25(78/1%)	209(98.6%)	
Atelectasis	Yes	5(15.6%)	10(4.7%)	**0.032**
	No	27(8.44%)	202(95.3%)	
CVA	Yes	1(0.5%)		**< 0.001**
	No	211(99.5%)		
FFP	Yes	15(46.9%)	33(15.6%)	**< 0.001**
	No	17(53.1%)	179(84.4%)	
PLT	Yes	11(34/4%)	31(14.6%)	**0.006**
	No	21(65.6%)	181(85.4%)	
P.C	Yes	21(65.6%)	103(48.6%)	0.072
	No	11(34/4%)	109(51.4%)	
Plural Efusion	Yes	10(31.3%)	17(8%)	**0.001**
	No	22(68.8%)	195(92%)	
AKI	Yes	13(40.6%)	30(142%)	**< 0.001**
	No	19(59.4%)	182(58.8%)	
Drainage	Below 1 lit	21(65.6%)	186(87.7%)	**0.003**
	Over 1 lit	11(34.4%)	26(12.3%)	
Cardiac dysfunction	Yes	6(18.8%)	11(5.2%)	**0.005**
	No	26(81.3%)	201(94.8%)	
Epinephrine (receiving days) (Mean ± SD)		0.84 ± 1.37	0.09 ± 0.41	**0.004**
Norepinephrine (receiving days) (Mean ± SD)		1.31 ± 1.69	0.29 ± 0.98	**0.002**
Dobutamine (receiving days) (Mean ± SD)		1.09 ± 1.91	0.39 ± 1.25	0.053
Milrinone (receiving days) (Mean ± SD)		0.25 ± 0.87	0.14 ± 0.71	0.418
Dopamine (receiving days) (Mean ± SD)		1.91 ± 2.57	0.81 ± 1.56	**0.024**
Hb (Mean ± SD)		13.51 ± 1.85	14.31 ± 2.14	0.049

Abbreviations: OR: odds ratio; BMI: Body Mass Index, EF: Ejection Fraction.,AKI: Acute Kidney Injury, CKD: Chronic Kidney Disease, CABG: Cronary Artery Bypass Graft, COPD: Chronic Obstractin Airway Disease.PC: Packed Cell, CVA(cerebrovascular accident)

**Table 2:** Relationship of demographic and clinical variables (before, during, and after surgery) with prolonged mechanical ventilation after surgery

Variables	Crude model		Adjusted Model	
Before surgery	OR (95% CI for OR)	P-value	OR (95% CI for OR)	P-value
Gender (Male vs. Female)	1.35 (0.62,2.94)	0.392	-	-
BMI	1.01 (0.93,1.11)	0.538	-	-
Age	1.02 (0.98,1.05)	0.245	1.02 (0.97,1.23)	0.432
EF	0.99 (0.96,1.03)	0.976	-	-
Underlying diseases				
Diabetes mellitus Yes vs. No	1.07 (0.52,2.23)	0.529	-	-
Hypertension Yes vs. No	1.11 (0.53,2.29)	0.581	-	-
CKD Yes vs. No	0.33 (0.09,1.17)	**0.061**	-	-
Urgency Yes vs. No	7.68 (2.74,9.88)	**< 0.001**	0.60 (0.29,0.81)	0.530
CABG Yes vs. No	1.02(0.36,2.79)	0.778	-	-
Copd Yes vs. No	2.02(0.62,6.57)	0.234		
EURO Score 2	1.47(1.15,1.89)	< 0.001	1.38(1.12,1.65)	0.038
**During surgery**				
Duration of surgery	1.01 (0.99,1.08)	0.280	-	-
Using pump Yes vs. No	0.49 (0.24,1.03)	0.096	0.84 (0.32,0.91)	0.861
Pump-time Yes vs. No	1.01 (1.01,1.06)	**0.036**	1.01 (0.95,1.14)	0.311
IABP Yes vs. No	3.82(1.49,9.80)	**0.018**	2.74(1.36,5.47)	0.031
**After surgery**			-	-
ICU admission (day)	1.66 (1.14,1.74)	**0.002**	1.53 (1.33,1.75)	**0.003**
Pneumonia Yes vs. No	6.75 (4.93,11.02)	**< 0.001**	7.24 (5.12,8.14)	**0.001**
Respiratory Failure Yes vs. No	8.34 (5.12,9.11)	**< 0.001**	7.56 (4.48,8.77)	**0.042**
Atelectasis Yes vs. No	2.97 (0.95,9.24)	**0.032**	1.54 (1.13,1.68)	0.660
CVA	8.11(4.39,11.13)	**< 0.001**	3.75(1.26,4.84)	**0.044**
FFP Yes vs. No	3.95 (1.87,8.34)	**< 0.001**	0.58 (0.48,0.71)	0.425
PLT Yes vs. No	2.30 (1.03,5.11)	**0.006**	1.96 (1.55,2.12)	0.301
P.C Yes vs. No	1.82 (0.89,3.71)	**0.072**	0.80 (0.68,0.97)	0.662
PE Yes vs. No	3.97 (1.65,9.54)	**0.001**	1.53 (1.16,1.77)	0.626
AKI Yes vs. No	3.56 (1.65,7.66)	**< 0.001**	1.08 (0.98,1.44)	0.918
Drainage Yes vs. No	2.82 (1.25,6.36)	**0.003**	2.23 (1.98,2.46)	**0.032**
Epinephrine (receiving days)	3.28 (1.89,2.56)	**0.004**	2.73 (2.24,3.11)	**0.022**
Norepinephrine (receiving days)	1.69 (1.31,2.21)	**0.002**	1.04 (0.89,1.19)	0.803
Dobutamine (receiving days)	1.32 (1.08,1.62)	0.053	1.15 (1.02,1.34)	0.295
Milrinone (receiving days)	1.17 (0.79,1.74)	**0.418**	0.80 (0.77,0.94)	0.381
Dopamine (receiving days)	1.23 (1.04,1.45)	**0.024**	0.86 (0.56,1.02)	0.298
Hb	0.81 (0.68,0.96)	**0.049**	0.84 (0.35,1.12)	0.128
Cardiac dysfunction Yes vs. No	4.22(1.44,8.32)	**0.005**	2.58(1.33,2.87)	0.042

Abbreviations: OR, odds ratio; BMI: Body Mass Index, EF: Ejection Fraction, CKD: Chronic Kidney Disease, CABG: Cronary Artery Bypass Graft. IABP: Intra Aortic Balloon Pump

## Discussion

The present study investigated factors contributing to the prolongation of mechanical ventilation(PMV) in patients after cardiac surgery from September 2021 to March 2022 in Shafa Hospital, Kerman. The findings suggested that the incidence of PMV after cardaic surgery was 13.1%. There was also a relationship between postoperative complications including pneumonia, respiratory failure, stroke after surgery, and high drainage with prolonged mechanical ventilation. Moreover, vasopressor epinephrine, cardiac dysfunctin, use of IABP and high EURO Score were associated with PMV. For each day of ICU stay, the chance of PMV increased by 1.5 times. In addition, ICU Mortality had a significant relationship with prolonged ventilation.(Pvalue < 0.001).

The findings from this study showed that the use of epinephrine, cardiac dysfunction, need to an IABP were associated with a 2-fold increase in the risk of PMV. Maybe that’s why the most common cause of PMV in urgent group was cardiac dysfunction.Koponen (2019) showed that the postoperative inotrope dose (vis max) is an independent predictor of adverse outcomes after cardiac surgery and an increased dose of vaspressors increases the length of stay and this index has a better-discriminating value compared to Apache 2, SAPS 2, and sofa score [9]. In this study, all inotropes were indexed together, but in the present study, among all inotropes, epinephrine was associated with an increased chance of PMV. Since epinephrine is the strongest inotrope, it can somehow justify the greater severity of heart failure, stunning myocardium, or shock in patients, leading to a PMV. Pinar Karaka (2021) reported that the most important risk factor for increased morbidity and mortality after cardiac surgery was the VIS score immediately after the operation, and it was considered more valuable than the Euro score [10]. Cardiac dysfunction in the first 30 days after the operation is one of the important causes of mortality after heart surgery [11, 12].

In the early postoperative period, it is essential to monitor low cardiac states and their treatment options. Temporary myocardial dysfunction may occur due to myocardial stunning and the inflammatory response triggered during heart surgery, typically resolving within 24 to 48 h. Effective management of these patients is crucial, as various types of shock—including hypovolemic, cardiogenic, distributive, and extracardiac obstructive shock—can arise following cardiac surgery [13].

The postoperative treatment plan, whether for fast tracking or not, is established in coordination with the surgeon and anesthesiologist, taking into account the patient’s surgical conditions, drainage volume, and hemodynamic status. Our study’s results support the correlation between cardiac dysfunction, the need for an intra-aortic balloon pump (IABP), and the use of the vasopressor epinephrine with PMV.

EUROSCORE 2 and the Society of thorasic surgens (STS) score are widely used in preoperative of risk assessment of cardiac operation [14].these evaluation systems could predict copmplications, hospitalization costs and long term prognosis [15, 16].

Similarly, we found that for each unit increase in EuroSCORE2, the likelihood of PMV increased by 1.38 times.

A higher preoperative severity of illness, as indicated by EuroSCORE2, is associated with PMV, highlighting the importance of thorough preoperative risk assessment and optimization of the patient’s condition.

In the present study, 13.1% of patients underwent prolonged ventilation. Mortality in patients requiring PMV after cardiac surgery was significantly higher than in patients without prolonged mechanical ventilation after cardiac surgery as confirmed by other studies [17–20]. Fernandes (2018) reported that 10–15% of patients admitted to ICU after cardiac surgery required mechanical ventilation for more than 48 h, and a high percentage of these patients died due to kidney and other organ failure and sepsis and The most common cause of death among patients who did not require PMVwas cardiogenic shock, which was more directly associated with the surgery and a history of heart disease [3]. While we did not specifically investigate the causes of mortality in our patient population, among those on PMV, three patients experienced sepsis and gastrointestinal bleeding, and three required dialysis. Additionally, it is well established that long-term ventilation increases the risk of ventilator-associated pneumonia (VAP). Main risk factors for ICUacquired.

BSI include high severity indexes at admission, prolonged stay, immunosuppression, liver disease, surgicaladmission, and the requirement for invasive devices or procedures [21].

Sankar reported the incidence of prolonged ventilation was 15% after risk matching and multivariate regression analysis. PMV was associated with surgical factors, including duration of surgery, history of heart surgery, pump failure, low EF, renal failure, and high pulmonary pressure. There was a strong correlation between postoperative complications and PMV and 30-day mortality [3]. The present study showed that postoperative complications including pneumonia, respiratory failure, stroke, and high drainage were associated with prolonged ventilation. Logically, these complications increase the duration of ventilation. In this research, the predominant factor contributing to PMV in elective surgery patients was identified as high drainage. Therefore, effective control of drainage during and after surgical procedures is crucial to mitigate this risk. Excessive bleeding following cardiac surgery correlates with increased rates of morbidity and mortality, as well as elevated healthcare costs.

Mediastinal bleeding can result from either surgical or medical factors. Surgical causes typically require revision, while medical causes include the use of antithrombotic medications, hypothermia, underlying diseases, and transfusion-related coagulopathy. These medical causes generally do not need surgical intervention. Management strategies can involve controlling coagulopathies, shivering maintaining stable blood pressure, to help reduce bleeding [13]. discontinuation of antiplatlet therapy for a time before surgery is warrented [22]. Due to the retrospective design of the study and insufficient data in patient records, it was not feasible to assess the use of antithrombotic medications among patients. Additionally, a portion of the patients underwent valve surgery, which increases the risk of bleeding due to anticoagulant use.

Pulmonary complications following cardiac surgery can include pneumonia (especially in patients requiring mechanical ventilation for over 24 h), respiratory failure, pleural effusion, atelectasis, pneumothorax, bronchospasm, aspiration pneumonitis, acute respiratory distress syndrome, and pulmonary embolism. These complications can lead to prolonged hospital stays and increased treatment costs [19]. In this study, pneumonia and respiratory failure were associated with (PMV).

Given that infection significantly contributes to morbidity and mortality surrounding surgical procedures, it is crucial to identify patients at risk. This allows for prompt control and treatment, particularly through the use of empirical antibiotic therapy tailored to the predominant microbial strains present in the ICU.

Contrary to findings in other literature, smoking and chronic obstructive pulmonary disease (COPD) did not emerge as predictive factors for (PMV) in our study. This may be attributed to effective treatment protocols, such as the use of nebulized bronchodilators and appropriate mechanical ventilation strategies for COPD patients, or it could reflect a low prevalence of COPD among our patient population. However, the retrospective nature of our analysis limits our ability to thoroughly assess these factors.

Michaud (2022) examined the predictor score for PMV and introduced independent variables such as heart failure before and after the operation, preoperative and postoperative lactate and preoperative creatinine greater than 4 upon entering the ICU, and the need for ECMO as the risk factors for prolonged ventilation [23]. Aksoy showed that the increased lactate level, long pump time, need for re-surgery to control bleeding, and preoperative leukocytosis were some independent risk factors for prolonged ventilation [24]. In the present study, it was not possible to check the association of lactate with prolonged mechanical ventilation.

Furthermore, patients who experienced a cerebrovascular accident faced more than a threefold increase in the likelihood of extended mechanical ventilation. Recent research indicates that major emboli originating from the ascending aorta, existing cerebral atherosclerotic lesions, or thromboembolic events occurring beyond significant carotid artery stenosis are the primary contributors to stroke during coronary artery bypass grafting (CABG) [25–27]. Additionally, carotid artery stenosis may serve as a surrogate indicator of widespread atherosclerotic disease rather than a direct cause. The presence of carotid artery stenosis has been linked to a notable rise in the risks of stroke within 30 days and mortality over 15 years. Therefore, screening with carotid duplex ultrasonography is strongly advised for high-risk patient populations based on the CHA2DS2-VASc score [28]. Effective management of arrhythmias and electrolyte imbalances is recommended to lower the risk of arytmia and ambolic stroke.

The findings of this study indicate that among elective patients, the primary cause of PMVwas drainage issues, while in the urgent group, cardiac dysfunction was the most prevalent cause. Statistical analysis revealed that factors such as cardiac dysfunction, the necessity for an intra-aortic balloon pump (IABP), high EuroSCORE, use of epinephrine, respiratory failure, and excessive drainage were independent risk factors for PMV.

To mitigate these risks, it is essential to enhance the cardiac and hemodynamic status of patients undergoing heart surgery. Additionally, careful management of drainage through proper surgical techniques and appropriate medical treatments, tailored to the patients’ histories of antithrombotic medication use, is recommended.

Another significant risk factor identified in this study was pneumonia and respiratory failure, highlighting the need for focused respiratory support post-open heart surgery. This includes respiratory physiotherapy, the use of mucolytics to facilitate effective secretion drainage, and pain management for these patients.

One of the strengths of the present study is the comprehensive investigation of possible risk factors before, during, and after cardiac surgery in PMV. Nonetheless, this study faced certain limitations due to its retrospective design, including gaps in data regarding antithrombotic agents and patient conditions during surgery. We suggest conducting multicenter studies with larger sample sizes that focus separately on valvular and urgent surgeries to yield more comprehensive results.

## Conclusion

The key findings of the study were that several postoperative factors were identified as independent risk factors for prolonged mechanical ventilation following cardiac surgery:

The primary predictive factors identified in this study included pneumonia, respiratory failure, postoperative stroke, excessive drainage, the use of the vasopressor epinephrine, cardiac dysfunction, reliance on an intra-aortic balloon pump (IABP), and a high EuroSCORE.

This research revealed that the findings from previous studies on predictors of prolonged mechanical ventilation have been conflicting, potentially due to differences in surgical techniques, postoperative care protocols, and patient populations. Therefore, further research with larger sample sizes and risk-matching approaches is needed to better elucidate the risk factors for prolonged ventilation in the context of cardiac surgery.

## Data Availability

No datasets were generated or analysed during the current study.
